# The Efficient Extraction Method of Collagen from Deteriorated Leather Artifacts

**DOI:** 10.3390/polym15163459

**Published:** 2023-08-18

**Authors:** Li Li, Meng Zhang

**Affiliations:** 1 Joint International Research Laboratory of Environmental and Social Archaeology, Institute of Cultural Heritage, Shandong University, Qingdao 266237, China; 2 Jining Museum, Jining 272145, China

**Keywords:** leather artifacts, collagen, extraction of ancient protein, conservation of cultural relics

## Abstract

Collagen is the most crucial component of leather artifacts and analyzing collagen can provide vital information for studying and conserving such artifacts. However, collagen in leather artifacts often faces challenges such as degradation, denaturation, and contamination, which make it difficult to achieve an ideal protein extract using traditional extraction methods. This study aimed to find an efficient collagen extraction strategy for aging leather by comparing and improving commonly used methods. The results of comparing different extraction methods indicated that a NaOH solution was highly effective in extracting collagen from aged leather. To determine the optimal conditions for collagen extraction from the NaOH solution, we conducted orthogonal experiments. The results revealed that a NaOH concentration of 0.05 mol/L, a dissolution temperature of 80 °C, and a dissolution time of 12 h were the most favorable conditions. To validate the effectiveness of this method, we performed SDS-PAGE and biological mass spectrometry tests on collagen extracts from leather samples with varying degrees of aging. All collagen extracts exhibited distinct bands in the gel, and the molecular weight of collagen in each sample exceeded 20 kDa. Furthermore, even with a reduced sample mass of 1 mg (micro-destructive sampling), biological mass spectrometry identified 124 peptides in the protein extract. Notably, four of these peptides were unique to cattle hide collagen and were not present in the collagen of pig, sheep, horse, deer, or human skins. These experimental findings confirm the efficacy of the NaOH solution for extracting collagen from aging leather, suggesting that it can serve as a significant method for collagen identification and analysis in leather artifacts.

## 1. Introduction

Leather was an important raw material for production in ancient societies. It played an important role in ancient military, clothing, and other fields [1,2]. The main raw material of leather is animal skin, which can easily decay and disappear in the process of burial [3,4]. Therefore, the leather relics excavated from archaeology are very precious and need rigorous scientific analysis and protection. Collagen is the main component of leather. Accurate identification of collagen can help precisely determine the source of ancient leather materials and reveal essential manufacturing details. Information regarding the structure and properties of collagen at the molecular level can assist in assessing the degree of leather aging and provide fundamental data for the formulation of conservation methods [5,6,7,8,9,10]. Therefore, analyzing collagen in leather artifacts holds crucial significance for their conservation.

According to archaeological findings, it is evident that the majority of leather artifacts unearthed in China exhibit significant deterioration and poor preservation conditions [7,11,12]. A large number of leather artifacts are severely degraded [6,11,12,13,14], making it difficult to carry out material identification, production process restoration, conservation, and other related research. Faced with this predicament, traditional leather analysis techniques, including infrared spectroscopy, thermogravimetric analysis, and microscopic morphological observation, etc. [14,15,16,17,18,19,20,21,22,23], face difficulties in accurately identifying and analyzing deteriorated leather artifacts. For example, a large number of interfering substances in the buried environment can be adsorbed in the cortical material, which can easily interfere with the detection accuracy of many characteristic peaks in the infrared spectrum of collagen. Severe degradation causes the micromorphological features of leather to almost disappear, which makes it difficult to obtain valid data for micromorphological observation [1,23].

New analysis technologies, with proteomics as a representative, have focused their research on the main component of leather collagen. Such techniques have played a significant role in material identification and the understanding of material degradation mechanisms. The enzyme-linked immunoassay (ELISA) based on immunology and the ZooMS and Bio-MS based on mass spectrometry are the main strategies of proteomics in ancient protein analysis at present [8,9,24,25,26]. It is crucial to highlight that the effective extraction of collagen from leather is a prerequisite for the application of the aforementioned techniques. However, as leather ages, the structure and properties of collagen in the artifacts may undergo changes, making conventional extraction methods not necessarily suitable for deteriorated leather artifacts. Hence, finding effective protein extraction methods is essential for the study and conservation of leather artifacts.

**Scheme 1:**
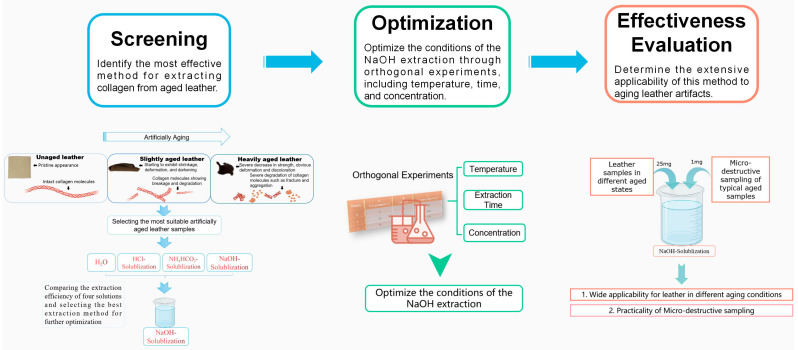
Screening, optimization, and testing of collagen extraction methods for deteriorated leather artifacts.

The commonly used methods for collagen extraction in modern industry and food industry include acid solubilization, enzyme solubilization, high-temperature water solubilization, etc. [27]. The commonly used methods for collagen extraction in archaeological samples include acid solubilization (HCl solution) and alkali solubilization (NH_4_HCO_3_ solution) [3,10,27,28]. These extraction methods have been applied in ancient animal bones, human bones, and a small amount of leather materials. To safeguard the invaluable leather artifacts, the sample size used for analysis is frequently minimal. However, enzymes, being proteins themselves, can act as interferences, potentially affecting subsequent analyses. The high-temperature water solubilization is a simple operation, but it has a low efficiency in extracting collagen. Hydrochloric acid, as a representative acidic solution, is a commonly used collagen extraction agent. However, many studies have also found that its extraction efficiency is not high. hydrochloric acid (HCl) solution and high-temperature water are unable to extensively disrupt the cross-linked structure of collagen. As a result, the efficiency of collagen extraction using these two methods is generally moderate. Leather artifacts frequently undergo prolonged aging, resulting in collagen degradation and structural changes, which may make the remaining collagen more challenging to dissolve [27,28,29]. Consequently, alkaline solutions with higher extraction efficiency are expected to yield superior results in extracting aged leather artifacts. To further screen and optimize the extraction method, we determined NaOH solubilization as the best extraction method based on the concept in Scheme 1. The optimal conditions for this method were obtained through orthogonal experiments. We verified the method’s wide applicability to aged leather artifacts and whether it meets the analysis requirements for micro-damaged sampling of cultural relics.

## 2. Materials and Methods

### 2.1. Materials and Reagents

The new vegetable tanned cattle leather was collected from the market (Houjie Dingtai Leather Company, Dongguan, China).

The required reagents for the artificial aging experiments include hydrochloric acid (HCl), ammonium bicarbonate (NH_4_HCO_3_), and sodium hydroxide (NaOH). All reagents are of analytical grade and were obtained from Sangon Biotech (Shanghai, China) Co., Ltd.

For the orthogonal experiments, the following reagents were used: NaOH (analytical grade, Sangon Biotech) and a protein concentration determination kit (Sangon Biotech).

The reagents used for electrophoresis experiments include precast gels, protein loading buffer, electrophoresis running buffer, protein maker, ethanol, methanol, acetic acid, ammonium sulfate, and Coomassie brilliant blue G250. All reagents are of analytical grade and were purchased from Sangon Biotech.

For the biomass spectrometry experiments, the required reagents include formic acid (chromatography grade, Sigma, St. Louis, MO, USA), acetonitrile (chromatography grade, Sigma), and trypsin (sequencing grade, Promega, Madison, WI, USA).

All reagents used in these experiments are of high purity and were procured from reputable suppliers. The quality of the reagents ensures the reliability and accuracy of the experimental results.

### 2.2. Methods

#### 2.2.1. Preparation of Aged Leather Samples

Aged archaeological leather artifacts experience collagen protein deterioration through two main pathways: de-tanning and degradation. Sodium hydroxide (NaOH) solution efficiently simulates these effects. In order to efficiently obtain artificially aged samples resembling the aged state of archaeological leather artifacts, high concentration of NaOH and reaction temperature were chosen for the experiment. The specific preparation process is outlined as follows:

The leather samples were cut into 5 × 5 cm squares. The cut samples were placed in 0.1 mol/L NaOH solution and aged at 60 °C for 6 h, 12 h, 24 h, 48 h. The aged leather samples were washed using distilled water. The cleaned samples were placed in pure water at 4 °C for storage [14,21,29,30,31].

The microscopic morphology and other characteristics of each aging sample were recorded by ultra-depth three-dimensional microscope (Smartzoom 5, ZEISS, Oberkochen, Germany).

#### 2.2.2. Extraction of Collagen from Aged Leather Samples

A total of 0.1 g of leather sample aged for 24 h was taken. The samples were ground and treated according to the parameters in Table 1. All experiments of this step were carried out in a 25 mL beaker. Each beaker was covered by aluminum foil and heated in a water bath.

The mass difference of the sample in various solutions was calculated according to the following equation. Compare the quality differences among the various methods and select the method with the largest quality difference for further optimization.


$$Mass\;difference=\frac{M1-M2}{M1}\times 100\%$$

*M*1, Total original mass of leather sample.

*M*2, Remaining mass of leather sample after extraction.

#### 2.2.3. Optimization of Experimental Conditions for the Extraction of Collagen from Aged Leather Samples by NaOH-Solublization (NS)

##### Making Bradford Standard Protein Concentration Curve

The Modified Bradford Protein Assay Kit was utilized to generate a protein concentration standard curve. The provided 1 mg/mL bovine serum standard protein solution in the kit was diluted with pure water to obtain different concentrations: 0 μg/mL, 50 μg/mL, 100 μg/mL, 150 μg/mL, 200 μg/mL, 250 μg/mL, and 300 μg/mL. The sample with 0 μg/mL concentration was used as the blank control, and the absorbance values (A595) of each tube were measured using a UV-Visible spectrophotometer (UV3000, Shanghai Jingke indusrtial, Shanghai, China). The absorbance values of each concentration sample were plotted on the *Y*-axis, and the corresponding protein concentrations were plotted on the *X*-axis to construct the standard curve. Based on this curve, the concentration of each collagen extract was tested.

##### Orthogonal Experiment

As shown in Table 2, the three factors of the orthogonal experiment were NaOH solution concentration, extraction temperature, and extraction time. Three levels were set for each factor.

A total of 25 mg of aged leather sample was taken for each test involved. The sample was added to a 10 mL centrifuge tube, and 3 mL of sodium hydroxide solution was added before placing it in a water bath for heating. The centrifuge tubes were all sealed with aluminum foil.

After extraction, the samples were purified and concentrated using the same method. The collagen extract was centrifuged at 10,000 r/min for 10 min. The supernatant was dialyzed for 24 h (the dialysis external solution was 500 mL of pure water, the pore size of the dialysis bag was 3000 Da, and the external solution was changed every 8 h). The dialyzed solution was centrifuged at 10,000 r/min for 10 min and the supernatant was filtered through a 0.45 μm filter membrane. Finally, the solution was concentrated to approximately 500 μL by ultrafiltration (3000 Da).

Extraction solution was diluted 10 times and then measured by the developed standard curve. The protein concentration in the extraction solution was used as the basis for determination, and the best extraction conditions for collagen in aged leather artifacts were screened using orthogonal experiments.

#### 2.2.4. Effectiveness Evaluation of the Modified Collagen Extraction Method

##### Collagen Extraction of Leather Samples with Different Aging Degrees

A total of 25 mg of leather samples aged for 6 h, 12 h and 24 h were taken. In addition, 10 mg of leather samples aged for 48 h was taken (only about 10 mg was retained after degradation and drying of one 5 × 5 cm leather sample). The optimal conditions obtained through orthogonal experiments were used for the collagen extraction of each sample. Referring to the method in Section 2.2.3, the protein extraction solutions were purified and concentrated. The protein concentrations of each extraction solution were determined using the established standard curve.

##### Collagen Extraction with Micro-Destructive Sampling

A total of 1 mg of leather samples aged for 24 h was taken. The optimal conditions obtained through orthogonal experiments were used for the collagen extraction. Referring to the method in Section 2.2.3, the protein extraction solutions were purified and concentrated. The protein concentrations were determined using the established standard curve.

##### SDS-PAGE

SDS-PAGE experiments were performed using the Laemmli method [32]. A total of 20 μL protein samples to be tested were mixed with 5μL Protein Loading Dye (Tris-HCl, pH 6.8; Glycerin; SDS; Bromophenol blue; and DTT), and then heated for denaturation. Subsequently, each mixture was added to precast gel (Precast-Glgel Tris-Glycine PAGE, BBI, Shanghai, China) and electrophoresed (PowerPacBasic, BioRad, Hercules, CA, USA) in Tris-Glycine SDS-PAGE Running Buffer (25 mM Tris, 192 mM Glycine, 0.1% SDS). Electrophoresis was conducted in constant voltage mode. The initial voltage was set at 60 V, and after 15 min, the voltage was increased to 120 V and maintained until the end of electrophoresis.

Following electrophoresis, gels were subjected to staining using the Blue Silver method [33]. The gel was immersed in fixative solution (10% acetic acid, 40% ethanol, 50% deionized water) for 30 min, followed by two washes with pure water for 15 min each. Subsequently, the washed gel was stained in staining solution (0.12% Coomassie brilliant blue G-250, 10% ammonium sulfate, 10% phosphoric acid, and 20% methanol) for 10–12 h. Finally, the gels were washed with pure water and the staining patterns of each lane were observed and recorded.

##### Biological Mass Spectrometry Analysis

A total of 30 μL Collagen extract by micro-collection was digested with 1 µg of sequencing grade modified trypsin at 37 °C for 16 h.

Identification of collagen peptides were performed by nanoLC-QE using a Q Exactive mass spectrometer (Thermo Fisher, Waltham, MA, USA) coupled to an Easy-nLC 1000 system (Thermo Fisher).

The enzymatically cleaved protein solution was subjected to the C18 column. The C18 column was equilibrated with 95% of solution A and the samples were loaded onto the Trap column by an autosampler. Peptides were eluted from the column using an acetonitrile gradient: 0 to 20 min, linear gradient of solution B from 4% to 50%; 20 min to 24 min, linear gradient of solution B from 50% to 100%; 24 min to 30 min, solution B maintained at 100%. Solution A was 0.1% formic acid in water and solution B was 0.1% aqueous formic acid in acetonitrile (84% acetonitrile) [24,25,26,33].

The mass-to-charge ratios of peptides and fragments of peptides were collected as follows: 10 fragmentation profiles (MS2 scan) were collected after each full scan.

Proteomics data files were searched using MaxQuant v2.1.4.0 against a local database. This database contained collagen sequences of most mammals download from NCBI (28 November 2021)

## 3. Results and Discussion

### 3.1. Preparation of Artificial Aging Samples

Alkaline aging is a common method used for artificial aging of leather, and sodium hydroxide (NaOH) solution is one of its representative agents. The NaOH solution not only facilitates the detachment of tanning agents bound to the collagens in leather but also leads to rapid degradation of collagen through alkali hydrolysis. Under appropriate concentrations and temperatures, sodium hydroxide can efficiently and conveniently obtain artificially aged leather samples with different aging degrees [21,29,30,31].

**Figure 1:**
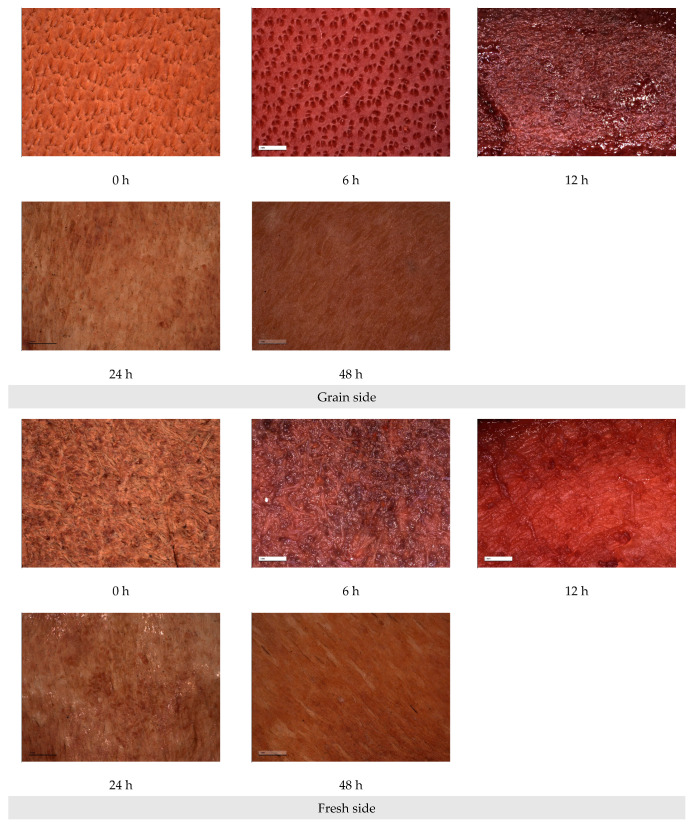
Microscopic morphological photographs of leather samples with different aging degrees.

Artificial aging experiments indicate that aging causes a series of morphological and performance changes in the leather, including color deepening, dehydration and hardening, curling, and shrinkage. These characteristic changes share many similarities with previous research and some unearthed leather artifacts in China [6,9,12,29]. The microscopic morphology of leather, such as the shape and distribution of pores in the leather grain side, is one of the important basis for the identification of leather types. However, alkaline aging also severely damages the microscopic morphology of the leather. Figure 1 illustrates the microscopic morphology of artificially aged leather samples in different states in this study. The microscopic morphology of the leather samples changed significantly as the aging time increased. In the unaged leather, clear pores and well-defined concave and convex structures formed by the expansion of pores can be observed on the grain surface. With the aggravation of aging, the pore morphology of the leather grain surface is gradually destroyed, and the concave–convex structure became less distinct. The pore characteristics of the grain side of the leather samples aged for 24 h showed considerable blurring. Upon extending the aging time to 48 h, the pore morphology of the leather grain side almost disappeared completely.

Furthermore, the unaged leather flesh side was characterized by dense fibers as its distinctive morphological feature. However, with increasing aging time, the fiber morphology of the leather flesh side gradually disappeared. Aging also causes a gradual reduction in the thickness of the leather. After aging the samples for 48 h, a substantial loss of components in the leather occurred, resulting in the transformation of the leather into a thin film with the complete disappearance of its morphological characteristics. In fact, it became challenging to distinguish between the flesh side and the grain side.

The excavated leather samples hold immense value due to their historical significance. Previous studies have documented various issues observed in these unearthed leather artifacts, such as a thin and brittle appearance, a lackluster color, shrinkage, deformation, and indistinct microscopic features [1,6,12,34,35,36,37]. Based on their similarity to the aged leather artifacts, the leather samples aged in NaOH solution for 24 h were selected as experimental samples for further analysis in this study.

### 3.2. Comparison of Collagen Extraction Methods from the Aging Leather Samples

**Figure 2:**
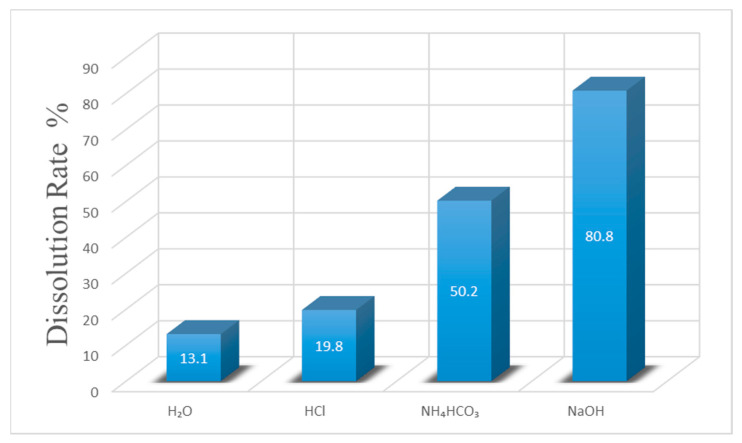
The mass difference of leather samples by different extraction methods.

The mass difference (MD) observed in aging leather samples before and after dissolution visually reflects the effectiveness of different collagen extraction methods. It can be seen from Figure 2 that significant variations in the MD of collagen were observed when using different dissolution methods for aging leather samples. The two methods of hydrochloric acid (HCl) solution and high-temperature pure water were less effective in extraction. The lowest mass difference of collagen was found in high-temperature water (only 13%). The mass difference in the HCl solution was less than 20%. The mass difference of aging leather samples was significantly increased in alkaline solutions. Ammonium bicarbonate (NH_4_HCO_3_) solution is a common method for the extraction of collagen from leather artifacts. The experimentally measured mass difference of this method was about 50%. The mass difference of sodium hydroxide (NaOH) solution was as high as 81%.

The significant differences observed among different extraction methods may be attributed to the nature of collagen in leather and the tanning and aging processes of leather. The collagen molecule is composed of three distinct alpha chains forming a triple-helix structure. Collagen fibers are stabilized by a wide molecular intermolecular cross-linked network, forming a stable spatial structure. To achieve efficient extraction of collagen, it is necessary to disrupt this cross-linked structure to some extent. However, hydrochloric acid (HCl) solution and high-temperature water are unable to extensively disrupt the cross-linked structure of collagen [27,28,29,38]. As a result, the efficiency of collagen extraction using these two methods is generally moderate. Nevertheless, in comparison to previous research, the extraction efficiency of collagen from aged leather using these two methods in this study is lower. This discrepancy may be related to the changes in collagen structure during the leather tanning, de-tanning, and aging processes.

Vegetable tanning is a well-established leather tanning technique with a rich history. Vegetable-tanned leather is essentially the product of raw hide collagen denaturation through multi-point hydrogen bonding with vegetable tannins [1,4]. This tanning process enhances various microstructures of collagen in raw hides, such as increasing collagen fiber structure porosity and reducing adhesion among collagen fiber bundles, fibers, and raw fibers. However, as leather artifacts age, the tanning agent gradually separates from the collagen, leading to the destruction of the spatial structure of the collagen in vegetable-tanned leather [1,14,23,29]. Collagen fiber bundles become intertwined and bonded to each other, resulting in decreased solubility. Under such circumstances, a high-temperature water and low-concentration HCl solution cannot easily rapidly disrupt the cross-bonded collagen fiber bundles, resulting in limited collagen dissolution [27,38].

Alkaline solutions can effectively disrupt the cross-linked structure of collagen, leading to an increased solubility of collagen. This results in a significant enhancement of the mass difference (MD) for the two alkaline solutions. Specifically, the MD of sodium hydroxide (NaOH) solution, being a strong alkali, exceeds 80%. NaOH solution can cleave the cross-links that hold the tropocollagen molecules together and enable the formation of collagen fibers with the aid of cross-links. Moreover, sodium hydroxide solution can induce partial degradation of collagen fibers in leather. The peptide bonds in collagen protein molecules, as well as the intermolecular and intramolecular cross-links formed during leather aging, can all be disrupted by sodium hydroxide solution. The degradation products of collagen are more easily dissolved into the solution. These factors collectively facilitate the dissolution of collagen in aged leather when treated with NaOH solution.

It should be noted that enzyme solubilization, a commonly used method for collagen extraction, was not included in the comparison. Due to the limited sampling amount of leather cultural relic samples, the enzymes themselves, being proteins, may interfere with subsequent analyses. Therefore, we believe that the enzyme solubilization may not be suitable for protein extraction of leather cultural relic samples.

The comparative analysis revealed that NaOH solution exhibited the most effective extraction of collagen from aged leather samples. Although NaOH solubilization may cause partial degradation of collagen, the extracted collagen degradation products still offer valuable information for material identification and the conservation of leather artifacts.

### 3.3. Optimization of Experimental Parameters for NaOH Extraction Method

Partial degradation of collagen facilitates the efficient preparation of protein solutions; however, excessive degradation may lead to a low molecular weight of collagen, which can be unsuitable for identification and analysis purposes. Therefore, in this study, a pore size of 3000 Da was selected during the dialysis and purification process to ensure that the extracted collagen maintained a sufficient molecular weight. The concentration of extracted collagen was used as a criterion to determine the optimal conditions (solution concentration, dissolution time, and dissolution temperature) for the extraction of collagen from aged leather by NaOH solubilization. As shown in Table 3, we designed a three-factor, three-level orthogonal experimental table containing extraction temperature, extraction time, and NaOH solution concentration. The effect of each factor on the protein concentration in the extraction solution was compared by orthogonal experiments, and the results are shown in Table 4.

The results of variance analysis showed that the optimal combination of factors in the NaOH solution extraction method was B2C2A2. Subsequent testing confirmed that the concentration of collagen extract obtained by this combination is 2692.75 μg/mL, which is higher than the experimental combination B2C2A1 with the highest protein concentration (2677.75 μg/mL) in the orthogonal experiment table. Therefore, it can be determined that B2C2A2 represents the best experimental combination, signifying the optimal conditions for collagen extraction using NaOH solubilization as follows: temperature 80 °C, extraction time 12 h, and sodium hydroxide concentration 0.05 mol/L.

### 3.4. Evaluation of the Effectiveness of NaOH Solubilization

An effective and superior method for ancient protein extraction should be capable of extracting collagen from leather samples at various aging stages, making them suitable for analysis. As shown in Table 4 (No. 1–4), NaOH solubilization can extract collagen from leather samples of all aging stages.

There are also some differences in the protein extraction efficiency among samples with different aging durations. The protein concentration in the extraction solutions of samples aged for 6 h and 12 h is lower than that of samples aged for 24 h. This could be attributed to the relatively short aging time, as the samples aged for 6 h and 12 h may not have completed full de-tanning yet. During the initial stages of interaction between these samples and the alkaline solution, the solution might still be actively de-tanning the collagen, which consumes a portion of the sodium hydroxide in the solution and affects collagen dissolution. Consequently, under the same conditions, samples aged for a shorter duration have a slightly lower collagen concentration due to this effect. As for the sample aged for 48 h, due to more severe aging, a significant amount of collagen may have undergone substantial degradation. The partial degradation caused by the NaOH solution during the dissolution process could further exacerbate the decrease in molecular weight of the remaining collagen molecules, potentially even falling below 3000 Da. As a result, the protein concentration in the extraction solution of the sample aged for 48 h is also slightly lower [1,29,31]. Overall, all the extraction solutions retained a considerable amount of collagen protein suitable for subsequent analysis.

Micro-destructive sampling is the development trend of cultural relic sample research. The ability to extract collagen from a minimal number of leather samples for subsequent analysis is crucial for scientific investigations on ancient leather artifacts. As shown in Table 4 (No. 5), the method still demonstrates excellent extraction efficacy even with small sample amounts. It can successfully extract collagen from aged leather samples with a sample mass as low as 1 mg. The resulting collagen solution can be utilized for various analyses, for example biological mass spectrometry. This method proves to be highly valuable for studying ancient leather samples with limited available material.

**Figure 3:**
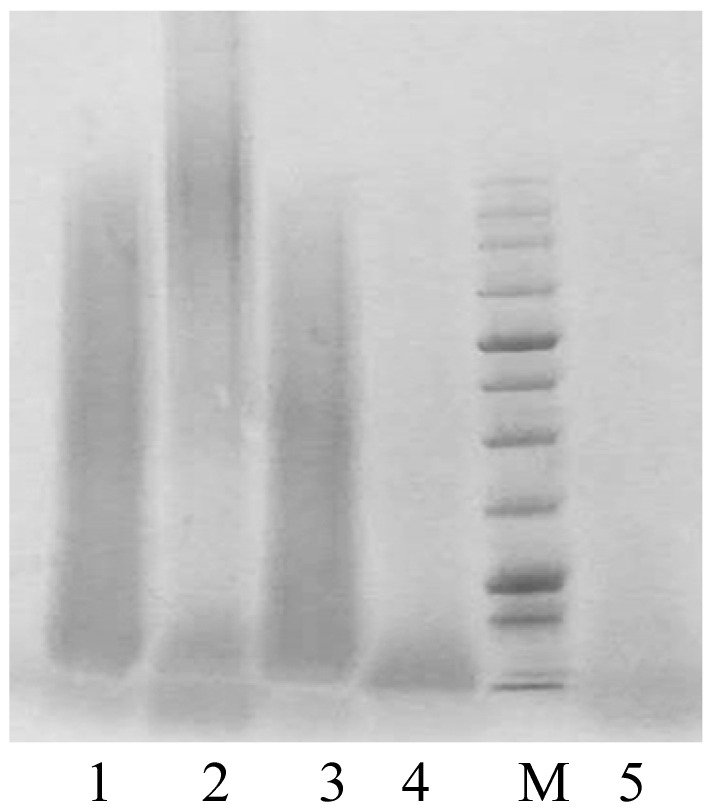
SDS-PAGE experimental results of each protein extract. Lanes 1–4 are the extracts of leather samples aged 6, 12, 24, and 48 h, respectively. Lane M is the standard protein. Lane 5 is the extract of the micro-collection sample.

SDS-PAGE is one of the basic methods of protein analysis. Each collagen extract was subjected to SDS-PAGE, and the obtained results aided in the initial assessment of their suitability for experimental analysis. Figure 3 shows the electrophoresis results of the collagen solutions from different aged leather samples. A wide distribution of protein bands in the gel would indicate that the extracted collagen suffered degradation during aging and the extraction process. As both leather aging and NaOH solubilization caused partial degradation of collagen, samples 1–4 exhibited diffuse protein distributions in the gel. With reference to the standard protein (lane M), some collagen molecules with large molecular weight were retained in each extract. These large molecular weight proteins (or peptides) are the main research materials for the subsequent scientific and technical analysis of leather artifacts such as leather material identification and the structure change pattern of collagen in aged leather.

**Figure 4:**
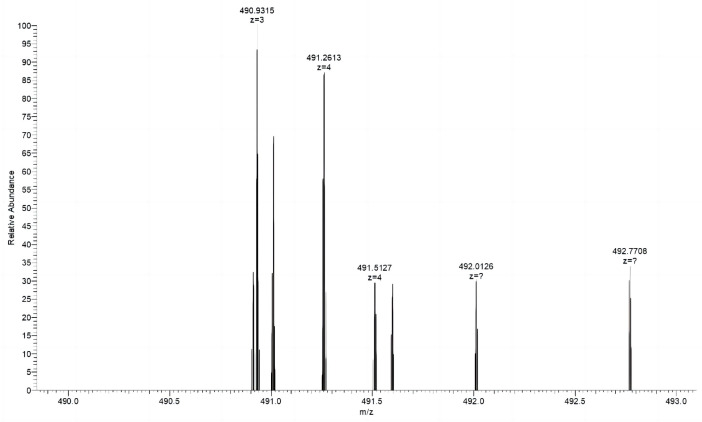
Full-scale scan of the first-class mass spectrum of the peptide VIDELDVKPEGTR.

Micro-destructive sampling plays a crucial role in the analysis and conservation of cultural relics. Figure 4 (lane 5) demonstrates that, even with a reduced sample mass of 1 mg, faint protein staining was still discernible in the lane, albeit with decreased intensity. Biological mass spectrometry serves as a significant approach for the identification and analysis of ancient proteins, offering high sensitivity, accuracy, and robust anti-interference capabilities. Leveraging these advantages, we can accurately identify protein components in micro-samples obtained through micro-destructive sampling.

**Figure 5:**
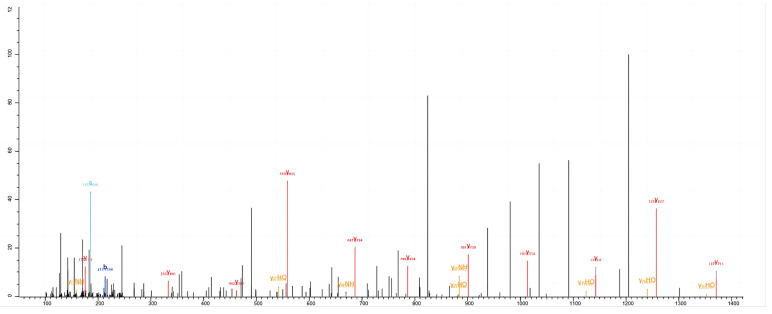
MS/MS spectrum and the b and y fragments of the peptide sequence VIDELDVKPEGTR.

As shown in Table 5, 124 different collagen peptides, such as AGERGVPGPPGAVGPAGK, DGEAGAQGPPGPAGPAGER, and GADGAPGK, were identified in the extract of the aging leather by micro-destructive sampling. We performed a comparative analysis by aligning all acquired peptide sequences with collagen peptide sequences derived from prominent ancient leather sources, including cattle, pig, buffalo, sheep, horse, deer, and human skin. The inclusion of human skin collagen sequences in this comparison aimed to account for potential contamination in the experimental samples. This meticulous comparison allowed us to discern the unique peptide profiles associated with each source and accurately identify the origin of the analyzed leather artifacts. As shown in Table 6, four peptides of the aging leather sample are unique to cowhide (they shared by cattle and buffalo skin collagen). Among them, the peptide VIDELDVKPEGTR is specific to cattle (*Bos taurus*) hide. As an example, the full-scale scan of the MS and the MS/MS spectrum of the peptide VIDELDVKPEGTR are shown in Figure 4 and Figure 5. The experimental results showed that rich peptide information could be retained in the micro-destructive sampling of an aging cowhide sample extracted by NaOH solubilization, which could satisfy the accurate identification of the hides origin.

The findings derived from both SDS-PAGE and biological mass spectrometry experiments substantiate the efficacy of NaOH solubilization for facilitating the micro-destructive sampling analysis of leather artifact samples. This robust approach successfully satisfies the requirements of protein extraction and identification, enabling comprehensive characterization and investigation of the examined artifacts.

## 4. Conclusions

The effective extraction of collagen is a prerequisite for many modern proteomic analysis techniques to be implemented. These techniques have gradually become important means for the analysis and conservation of ancient artifacts made by protein materials. Prolonged aging induces structural and property changes in collagen within leather artifacts, leading to suboptimal results with many conventional collagen extraction methods. It necessitates the reevaluation and optimization of collagen extraction methods specifically tailored for aged leather artifact samples. Among various collagen extraction methods, alkaline solution exhibits distinct advantages, with a remarkable mass difference of over 80% for aged leather in NaOH solution. The optimal extraction conditions for collagen in aging leather artifacts using NaOH solubilization were determined through an orthogonal experiment. The optimized parameters included a NaOH concentration of 0.05 mol/L, a dissolution temperature of 80 °C, and a dissolution time of 12 h. These precise conditions were ascertained to ensure efficient and effective extraction of collagen from the aged leather samples.

The extensive applicability of the NaOH solubilization method is demonstrated through SDS-PAGE analysis of collagen extracts from leather samples exhibiting varying degrees of aging. Remarkably, even with a reduced sampling amount of 1 mg, the protein extract not only successfully completes the SDS-PAGE experiment but also yields a wealth of collagen peptide information in subsequent biological mass spectrometry analysis. The accurate identification of the collagen extract as originating from cattle hides further strengthens the validity of the approach.

The comprehensive experimental outcomes collectively highlight the efficacy of the NaOH-solubilization method in extracting collagen from diverse leather artifacts while satisfying the subsequent analytical requirements. The results firmly establish the potential of this method to significantly contribute to the advancement of collagen identification and analysis in the realm of leather artifacts.

## Figures and Tables

**Table 1:** Experimental parameters of each collagen extraction method.

No.	Method	Temperature/°C	Time/h	Volume/mL
1	0.1 mol/L NaOH	80	12	10
2	0.01 mol/L HCl	80	12	10
3	0.05 mol/L NH_4_HCO_3_	80	12	10
4	Water	100	12	10

**Table 2:** Experimental parameters of each collagen extraction method.

Factors.	A (Temperature/°C)	B (Time/h)	C (Concentration/mol/L)
1	70	9	0.01
2	80	12	0.05
3	90	15	0.10

**Table 3:** Results of orthogonal experiments.

No.	A (Temperature/°C)	B (Time/h)	C (Concentration/mol/L)	Protein Concentration μg/mL
1	1 (70)	1 (9)	1 (0.01)	560
2	1 (70)	2 (12)	2 (0.05)	2678
3	1 (70)	3 (15)	3 (0.10)	1590
4	2 (80)	1 (9)	2 (0.05)	2329
5	2 (80)	2 (12)	3 (0.10)	1145
6	2 (80)	3 (15)	1 (0.01)	2022
7	3 (90)	1 (9)	3 (0.10)	1398
8	3 (90)	2 (12)	1 (0.01)	1794
9	3 (90)	3 (15)	2 (0.05)	1917
K1	1609	1429	1459	
K2	1832	1872	2308	
K3	1703	1843	1378	
Variance	223	443	930	

**Table 4:** The protein solution concentration of each aging leather samples.

No.	Aging Time (h)	Weight (mg)	Extract Concentration (μg/mL)	Protein Extraction Rate * (%)
1	6	25	1942	3.88
2	12	25	1489	2.98
3	24	25	2693	5.39
4	48	10	647	3.24
5	24	1	102	5.10

* Calculated percentage of total protein mass in each protein extraction solution relative to the total sample mass based on protein concentration.

**Table 5:** The peptides identified in the collagen extract by micro-destructive sampling.

Organism	Protein IDs	Protein	Total Peptides
Bos taurus	sp|P02453.3|CO1A1_BOVIN; NP_001029211.1; DAA18573.1; AAI05185.1; XP_024835395.1	Collagen alpha-1(I) chain	30
	NP_001068594.1	collagen alpha-2(VI) chain precursor	7
	sp|P02465.2|CO1A2_BOVIN	Collagen alpha-2(I) chain	17
	sp|P21793.2|PGS2_BOVIN	Decorin	10
	XP_002697097.2	collagen alpha-1(VII) chain	3
	NP_001106695.1	collagen alpha-1(II) chain isoform 2 precursor	4
	XP_024846034.1	collagen alpha-3(VI) chain isoform X5	25
	NP_001137337.1	collagen alpha-1(VI) chain precursor	9
	prf||0910139A; AAA30436.2	collagen alpha1(I)CN8	6
	XP_005202110.1	collagen alpha-2(VI) chain isoform X1	10
	XP_024835542.1	collagen alpha-2(V) chain	3

**Table 6:** The specific peptides obtained by sequence alignments in the collagen extract by micro-destructive sampling.

	Sequences
Specific peptides shared by cattle and buffalo skin collagen	IGQPGAVGPAGIR
	SGDRGETGPAGPAGPIGPVGAR
	DGRIGQPGAVGPAGIR
Peptide sequences unique to cattle skin collagen	VIDELDVKPEGTR

## Data Availability

All data other than the mass spectrometry raw data have been incorporated into the article.
